# Erratum to “Characteristics and prognosis of Japanese colorectal cancer patients: The BioBank Japan Project” [J Epidemiol 27(3S) (2017) S36–S42]

**DOI:** 10.1016/j.je.2017.04.001

**Published:** 2017-06-05

**Authors:** Akiko Tamakoshi, Koshi Nakamura, Shigekazu Ukawa, Emiko Okada, Makoto Hirata, Akiko Nagai, Koichi Matsuda, Yoichiro Kamatani, Kaori Muto, Yutaka Kiyohara, Zentaro Yamagata, Toshiharu Ninomiya, Michiaki Kubo, Yusuke Nakamura, BioBank Japan Cooperative Hospital Group, Wataru Ono, Hiromasa Harada, Shunji Kawamoto, Nobuaki Shinozaki, Shiro Minami, Takeshi Yamada, Hideyuki Suzuki, Kazuhiro Sakamoto, Kazuo Kaneko, Shinichi Ohba, Satoshi Asai, Mitsuhiko Moriyama, Yasuo Takahashi, Tomoaki Fujioka, Wataru Obara, Seijiro Mori, Hideki Ito, Satoshi Nagayama, Yoshio Miki, Akihide Masumoto, Akira Yamada, Yasuko Nishizawa, Ken Kodama, Tomoharu Shimizu, Shigeyuki Naka, Yukihiro Koretsune, Mitsugu Sekimoto, Hiroyuki Kokuto

**Affiliations:** m Tokushukai Hospitals, Japan; n Nippon Medical School, Japan; o Juntendo University, Japan; p Nihon University, Japan; q Iwate Medical University, Japan; r Tokyo Metropolitan Institute of Gerontology, Japan; s The Cancer Institute Hospital of JFCR, Japan; t Aso Iizuka Hospital, Japan; u Osaka Medical Center for Cancer and Cardiovascular Diseases, Japan; v Shiga University of Medical Science, Japan; w National Hospital Organization, Osaka National Hospital, Japan; x Fukujuji Hospital, Japan; a Department of Public Health, Hokkaido University Graduate School of Medicine, Sapporo, Japan; b Laboratory of Genome Technology, Institute of Medical Science, The University of Tokyo, Tokyo, Japan; c Department of Public Policy, Institute of Medical Science, The University of Tokyo, Tokyo, Japan; d Laboratory of Molecular Medicine, Institute of Medical Science, The University of Tokyo, Tokyo, Japan; e Laboratory of Clinical Genome Sequencing, Graduate School of Frontier Sciences, The University of Tokyo, Tokyo, Japan; f Laboratory for Statistical Analysis, RIKEN Center for Integrative Medical Sciences, Yokohama, Japan; g Hisayama Research Institute for Lifestyle Diseases, Fukuoka, Japan; h Department of Health Sciences, University of Yamanashi, Yamanashi, Japan; i Department of Epidemiology and Public Health, Graduate School of Medical Sciences, Kyushu University, Fukuoka, Japan; j RIKEN Center for Integrative Medical Sciences, Yokohama, Japan; k Section of Hematology/Oncology, Department of Medicine, The University of Chicago, Chicago, USA

Some numbers regarding the stage of overall rectal cancer patients and year of diagnosis of newly diagnosed rectal cancer patients in the original Table 1 were incorrect. The authors apologize for the error.

**Table 1:** Characteristics of colorectal cancer patients in BBJ.

	Overall patients						Patients registered within 90 days after diagnosis					
	Colorectal cancer		Colon cancer		Rectal cancer		Colorectal cancer		Colon cancer		Rectal cancer	
	N	(%)	N	(%)	N	(%)	N	(%)	N	(%)	N	(%)
Total	5864		3334		1893		1708		1018		517	
Sex												
Men	3699	(63.1)	2059	(61.8)	1269	(67.0)	1052	(61.6)	599	(58.8)	355	(68.7)
Women	2165	(36.9)	1275	(38.2)	642	(33.9)	656	(38.4)	419	(41.2)	162	(31.3)
Year of diagnosis												
2000	1287	(21.9)	691	(20.7)	436	(23.0)	–	–	–	–	–	–
2001	393	(6.7)	225	(6.7)	126	(6.7)	–	–	–	–	–	–
2002	529	(9.0)	300	(9.0)	164	(8.7)	–	–	–	–	–	–
2003	778	(13.3)	444	(13.3)	264	(13.9)	142	(8.3)	90	(8.8)	49	(9.5)
2004	852	(14.5)	508	(15.2)	261	(13.8)	348	(20.4)	218	(21.4)	105	(20.3)
2005	754	(12.9)	429	(12.9)	261	(13.8)	370	(21.7)	208	(20.4)	130	(25.1)
2006	657	(11.2)	387	(11.6)	204	(10.8)	395	(23.1)	245	(24.1)	107	(20.7)
2007	584	(10.0)	331	(9.9)	170	(9.0)	423	(24.8)	238	(23.4)	119	(23.0)
2008	30	(0.5)	19	(0.6)	7	(0.4)	30	(1.8)	19	(1.9)	7	(1.4)
Duration between diagnosis and registration												
- 90 days	1708	(29.1)	1018	(30.5)	517	(27.3)	1708	(100.0)	1018	(100.0)	517	(100.0)
- 0.5 years	631	(10.8)	347	(10.4)	226	(11.9)	–	–	–	–	–	–
- 1 year	616	(10.5)	359	(10.8)	193	(10.2)	–	–	–	–	–	–
- 2 years	801	(13.7)	449	(13.5)	270	(14.3)	–	–	–	–	–	–
- 3 years	489	(8.3)	282	(8.5)	158	(8.3)	–	–	–	–	–	–
- 4 years	348	(5.9)	194	(5.8)	102	(5.4)	–	–	–	–	–	–
- 5 years	268	(4.6)	147	(4.4)	91	(4.8)	–	–	–	–	–	–
- Longer than 5 years	1003	(17.1)	538	(16.1)	336	(17.7)	–	–	–	–	–	–
Stage												
0	71	(5.1)	46	(5.3)	24	(4.7)	13	(4.1)	9	(4.4)	4	(3.6)
I	256	(18.5)	140	(16.2)	114	(22.2)	62	(19.4)	38	(18.4)	21	(18.8)
II	394	(28.4)	269	(31.1)	122	(23.7)	87	(27.3)	57	(27.7)	31	(27.7)
IIIa	345	(24.9)	209	(24.2)	136	(26.5)	71	(22.3)	46	(22.3)	27	(24.1)
IIIb	108	(7.8)	67	(7.8)	41	(8.0)	17	(5.3)	13	(6.3)	4	(3.6)
IV	213	(15.4)	133	(15.4)	77	(15.0)	69	(21.6)	43	(20.9)	25	(22.3)
No information	4477	–	2470	–	1379	–	1389	–	812	–	405	–
Histology												
Adenocarcinoma	4037	(95.3)	2355	(95.2)	1345	(95.3)	1212	(95.5)	742	(95.6)	385	(95.1)
Adenosquamous carcinoma	15	(0.4)	9	(0.4)	4	(0.3)	6	(0.5)	3	(0.4)	1	(0.2)
Basaloid cell carcinoma	4	(0.1)	1	(0.0)	3	(0.2)	2	(0.2)	1	(0.1)	1	(0.2)
Squamous cell carcinoma	14	(0.3)	2	(0.1)	5	(0.4)	4	(0.3)	1	(0.1)	2	(0.5)
Carcinoid tumor	7	(0.2)	2	(0.1)	5	(0.4)	3	(0.2)	1	(0.1)	3	(0.7)
Malignant melanoma	2	(0.0)	0	(0.0)	2	(0.1)	0	(0.0)	0	(0.0)	0	(0.0)
Non-epithelial tumor	4	(0.1)	2	(0.1)	2	(0.1)	1	(0.1)	0	(0.0)	1	(0.2)
Lymphoma	1	(0.0)	1	(0.0)	0	(0.0)	0	(0.0)	0	(0.0)	0	(0.0)
Unclassified tumor	9	(0.2)	7	(0.3)	2	(0.1)	2	(0.2)	0	(0.0)	1	(0.2)
Metastatic tumor	7	(0.2)	5	(0.2)	2	(0.1)	1	(0.1)	1	(0.1)	0	(0.0)
Others	61	(1.4)	41	(1.7)	17	(1.2)	19	(1.5)	14	(1.8)	5	(1.2)
Unknown	77	(1.8)	48	(1.9)	24	(1.7)	19	(1.5)	13	(1.7)	6	(1.5)
No information	1626	–	861	–	482	–	439	–	242	–	112	–
History of Type 2 diabetes												
Absence/no information	5195	–	2946	–	1661	–	1532	–	913	–	459	–
Presence	669	(11.4)	388	(11.6)	232	(12.3)	176	(10.3)	105	(10.3)	58	(11.2)
Family history of colorectal cancer												
Absence/no information	5081	–	2849	–	1643	–	1472	–	868	–	442	–
Presence	783	(13.4)	485	(14.5)	250	(13.2)	236	(13.8)	150	(14.7)	75	(14.5)
Meat consumption												
Almost everyday	509	(9.6)	281	(9.0)	190	(10.8)	177	(11.2)	108	(11.1)	55	(11.2)
3–4 days/week	1678	(31.7)	1006	(32.2)	543	(30.8)	525	(33.3)	318	(32.8)	167	(33.9)
1–2 days/week	2317	(43.8)	1363	(43.7)	781	(44.3)	673	(42.7)	418	(43.1)	210	(42.6)
Almost never	789	(14.9)	472	(15.1)	249	(14.1)	202	(12.8)	125	(12.9)	61	(12.4)
No information	571	–	212	–	130	–	131	–	49	–	24	–
Green leafy vegetable consumption												
Almost every day	4153	(78.6)	2483	(79.6)	1348	(76.5)	1210	(76.6)	753	(77.5)	365	(73.7)
3–4 days/week	682	(12.9)	387	(12.4)	251	(14.3)	209	(13.2)	124	(12.8)	71	(14.3)
1–2 days/week	334	(6.3)	193	(6.2)	114	(6.5)	116	(7.3)	71	(7.3)	41	(8.3)
Almost never	114	(2.2)	56	(1.8)	48	(2.7)	45	(2.8)	23	(2.4)	18	(3.6)
No information	581	–	215	–	132	–	128	–	47	–	22	–
BMI												
<18.5	621	(11.2)	337	(10.7)	202	(11.4)	245	(14.8)	144	(14.7)	68	(13.5)
18.5–24.9	3813	(69.0)	2176	(69.1)	1216	(68.9)	1135	(68.7)	673	(68.9)	350	(69.6)
25–29.9	985	(17.8)	568	(18.0)	315	(17.8)	242	(14.7)	140	(14.3)	79	(15.7)
≥30	111	(2.0)	68	(2.2)	32	(1.8)	29	(1.8)	20	(2.0)	6	(1.2)
No information	334	–	185	–	128	–	57	–	41	–	14	–
Smoking status												
Never-smoker	2509	(43.6)	1490	(45.5)	706	(38.1)	724	(42.7)	464	(46.0)	176	(34.4)
Ex-smoker	2090	(36.3)	1201	(36.7)	749	(40.4)	638	(37.7)	367	(36.4)	226	(44.1)
Current smoker	997	(17.3)	558	(17.1)	383	(20.6)	297	(17.5)	171	(16.9)	107	(20.9)
Smoker with unknown status	162	(2.8)	23	(0.7)	17	(0.9)	35	(2.1)	7	(0.7)	3	(0.6)
No information	106	–	62	–	38	–	14	–	9	–	5	–
Alcohol intake												
Never-drinker	2396	(41.7)	1390	(42.6)	729	(39.4)	705	(41.7)	439	(43.7)	199	(38.9)
Ex-drinker	702	(12.2)	415	(12.7)	246	(13.3)	215	(12.7)	133	(13.2)	73	(14.3)
Current drinker consuming 0–15 g alcohol/day	999	(17.4)	611	(18.7)	339	(18.3)	257	(15.2)	157	(15.6)	82	(16.0)
Current drinker consuming 15–30 g alcohol/day	523	(9.1)	292	(9.0)	176	(9.5)	149	(8.8)	85	(8.5)	49	(9.6)
Current drinker consuming ≥30 g alcohol/day	903	(15.7)	509	(15.6)	324	(17.5)	314	(18.6)	182	(18.1)	102	(19.9)
Drinker with unknown status	218	(3.8)	45	(1.4)	36	(1.9)	50	(3.0)	9	(0.9)	7	(1.4)
No information	123	–	72	–	43	–	18	–	13	–	5	–
Physical exercise												
≥3 times/week	1183	(22.8)	704	(23.0)	366	(21.2)	298	(19.2)	182	(19.2)	85	(17.4)
1–2 times/week	229	(4.4)	137	(4.5)	74	(4.3)	54	(3.5)	33	(3.5)	19	(3.9)
No habit	3776	(72.8)	2215	(72.5)	1283	(74.5)	1197	(77.3)	733	(77.3)	384	(78.7)
No information	676	–	278	–	170	–	159	–	70	–	29	–

BBJ, BioBank Japan; BMI, body mass index.

